# Some Curious Patents

**Published:** 1890-10-25

**Authors:** 


### Some Curious Patents.

The law under which patents are granted in the United States directs that they may be issued for new and useful inventions. Very soon after the passage of the law it became necessary to decide as to what should be held to constitute a "useful" invention within the meaning of the Act, and after much deliberation it was ruled that anything should be considered "useful," from the patent point of view, provided that it was not detrimental to the public interests. For instance, you may patent an artificial leg for a frog, but you may not patent an appliance for preventing conception. This ruling has permitted the issue of some very curious patents, especially in the department of medicine and surgery. Patents for trusses, suspensories, syringes, surgical knives, pessaries, splints, etc., there are by the score. One inventor has given his attention to bunion protectors, another to poultice holders. There is a patent tape-worm trap, consisting of a gold capsule about the shape and size of the gelatine capsules used by pharmacists. This capsule opens longitudinally and sets with a spring, like an old-fashioned rat-trap, and has a ring at one end, to which a piece of silk thread is to be fastened. The patient is to fast two or three days, and then the trap is set, baited with a bit of cheese, and swallowed, leaving the silk string hanging out of the mouth. In a few moments it is supposed that the hungry worm will make a dash for the cheese, set free the spring, and leave the jaws of the capsule clasped about his head. Then by means of the string he is to be drawn out hand over hand and coiled away in a bottle, after which you may go fishing for another one. Then there is a complicated-looking piece of machinery which is described as "an apparatus to be used by surgeons in such operations where it is necessary or advantageous to have a practical operative microscope concentrated in the place to be operated on—such as the interior of the mouth, throat," and other passages. It is probably intended that the surgeon shall thus see the cells and fibres which he is dividing, decide whether they contain pus, cocci, or tubercle bacilli, or cancer nests, and govern his operations accordingly.

Most of the States have no anatomy laws, and the numerous medical schools have to supply material for their dissecting rooms as best they can.

The tales of grave robbing which often appear in the daily press have stimulated a number of inventors to provide ways and means for defeating the resurrectionists. There is a patent luminous ghost, which is to be placed in the coffin, and which will scare the robber into fits. There is a coffin torpedo, which will shoot anyone who attempts to open the grave; and a dynamite bomb, which will send the robber up and the coffin down, so that they are not likely to come together again in good condition.

A nose im prover has been patented, and also a cheek beautifier, the former being a sort of mould into which the nose is to be pressed every night until it assumes the desired Grecian or Roman contour; the latter a set of springs to be worn in the mouth to stretch out the wrinkles, and keep the cheeks plump.

Smokers who have sore teeth may have a patent pipe with a chin brase, upon which the weight of the pipe may rest; and the number of patent pipes is enormous.

